# Supplementary material for: Mapping the heritability of disease: a nationwide study
Source: Nat Commun. 2026 Mar 17;17:4080. doi: 10.1038/s41467-026-69991-z (PMC13144670; doi:10.1038/s41467-026-69991-z)
Supplement: Supplementary file 1 — Supplementary Information [file 41467_2026_69991_MOESM1_ESM.pdf]

Mapping the Heritability of Disease: A Nationwide Study

Supplementary Data

Supplementary Data 1 contains all twin estimates from both the 1955 and 1977 birth cohorts with standard errors, 95% confidence intervals, r_{ss} and r_{os}, uncorrected and corrected p values, onset type, and functional domain.

Supplementary Data 2 contains all sibling estimates from both the 1955 and 1977 birth cohorts with the same variables as Supplementary Data 1.

Supplementary Data 3 contains snp-h^2, polygenicity, and selection coefficients with 95% confidence intervals for 10 brain disorders.

Supplementary Data 4 contains sex ratios for both the 1955 and 1977 sibling and twin cohorts.

Supplementary Figures

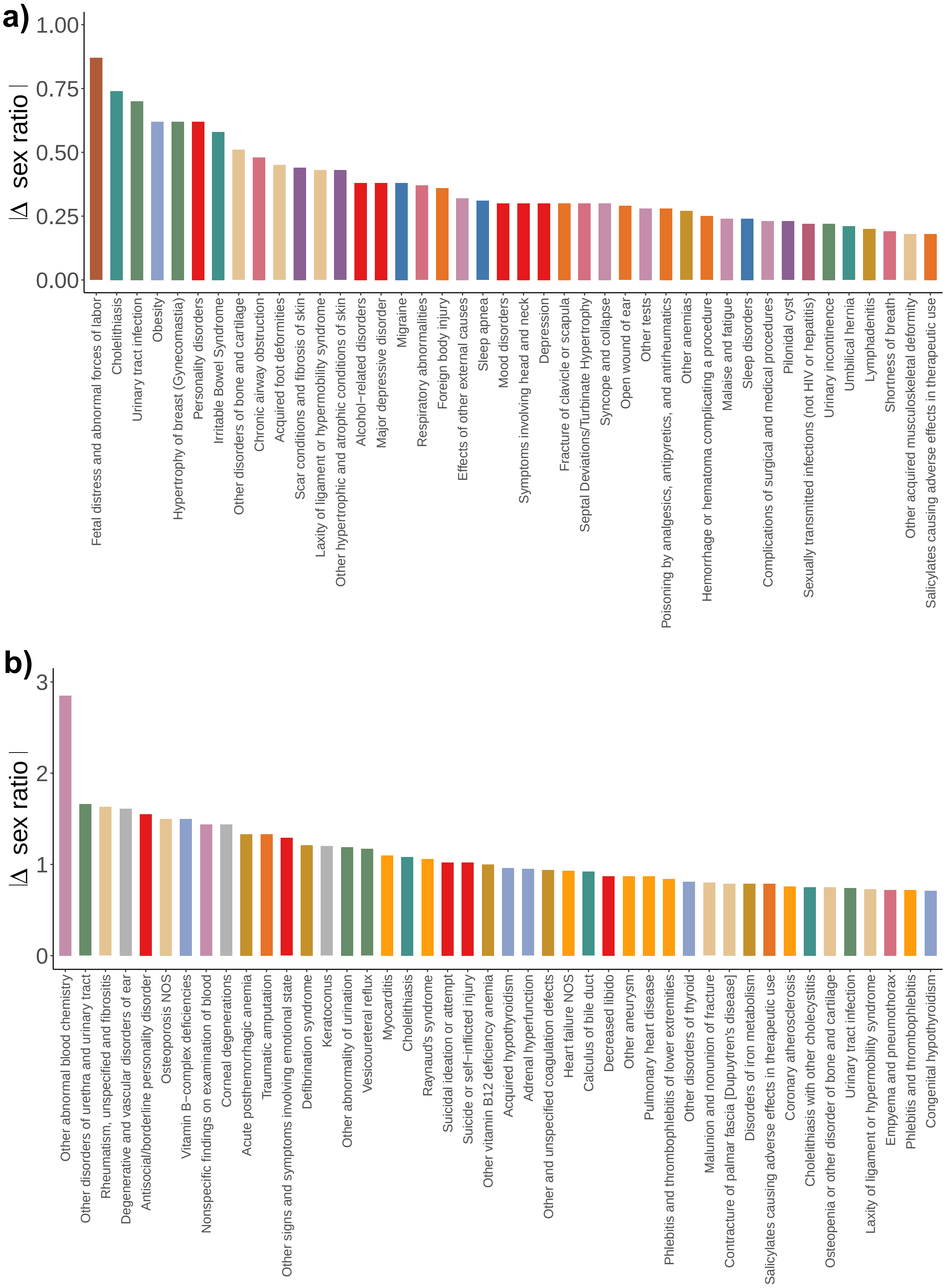

Supplementary Figure 1: Phenotypes with largest differences in sex ratios. Phenotypes with largest absolute differences in sex ratios between the 1977 and 1955 birth cohort for (a) the twins and (b) all siblings.

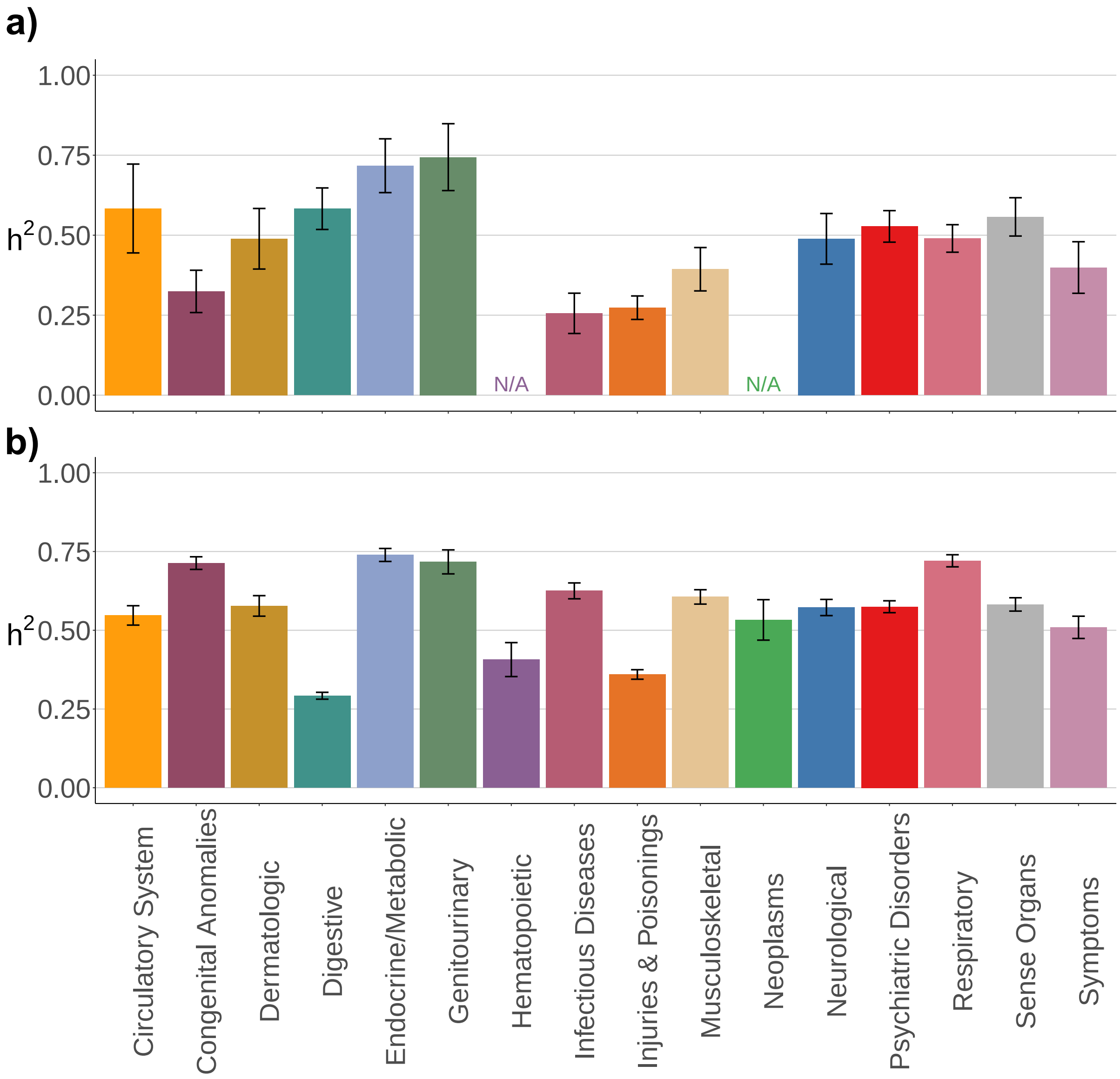

Supplementary Figure 2: Inverse variance weighted mean heritability (h^2) on the liability scale across functional domains. Distribution of the weighted mean liability-scale heritability estimates (h^2) for each high-level phecode-derived disorder group, based on the (a) 1955 twin birth cohort and the (b) 1955 sibling cohort. Error bars denote 95% confidence intervals.

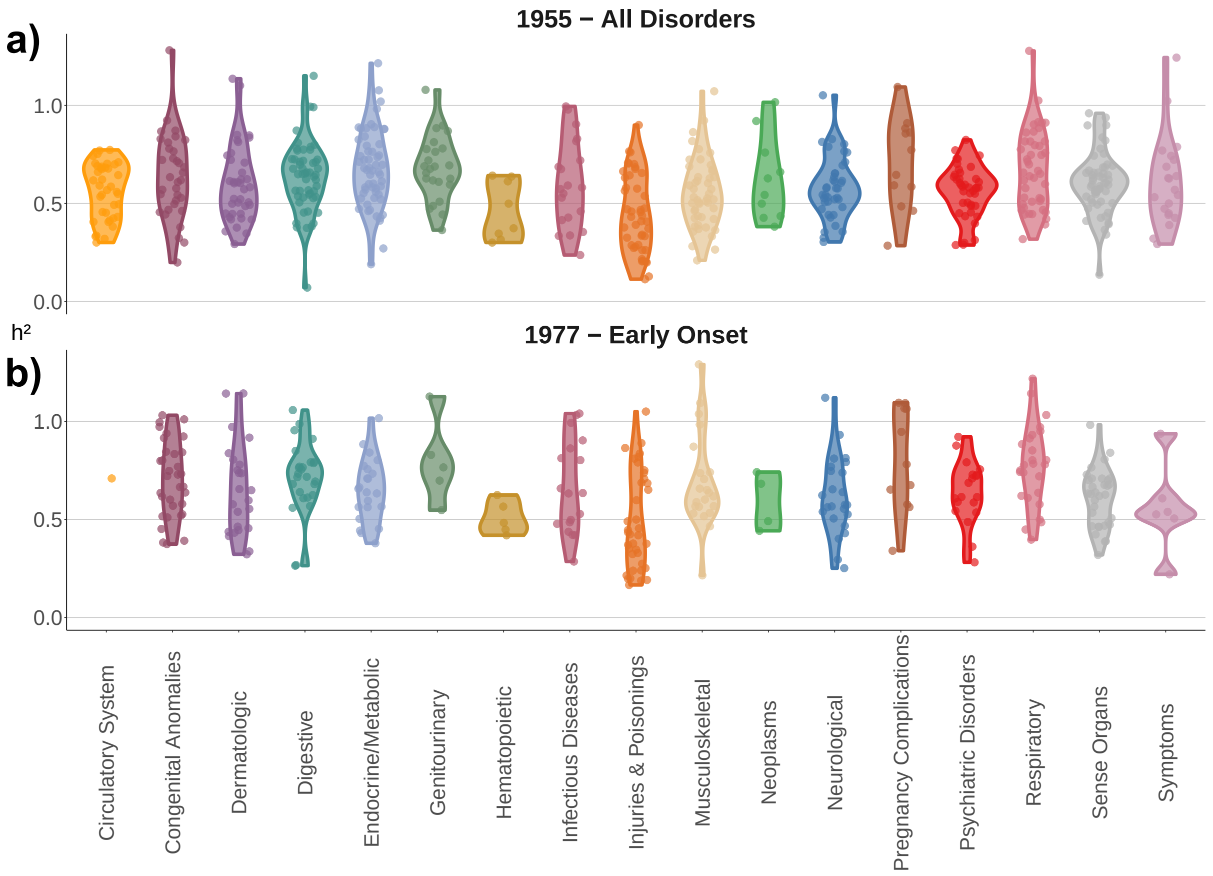

Supplementary Figure 3: Sibling heritability estimates across functional domains and birth cohorts. Distribution of liability-scale heritability estimates across functional domains, stratified by onset classification. Panel (a) includes all phenotypes from the 1955 sibling cohort irrespective of onset classification, while panel (b) presents early-onset phenotypes estimated from the 1977 birth cohort.

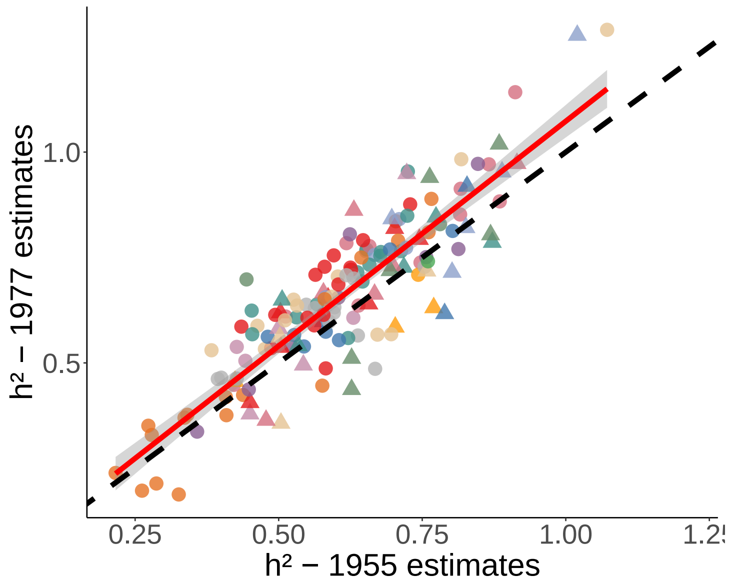

Supplementary Figure 4: Comparison of sibling heritability estimates for phenotypes in the 1955 and 1977 birth cohorts. Each point represents a phenotype, coloured according to its functional domain and shaped by onset classification (circle = early onset; triangle = late onset). The red line indicates the linear regression fit, while the dashed black line represents the identity line (slope = 1).

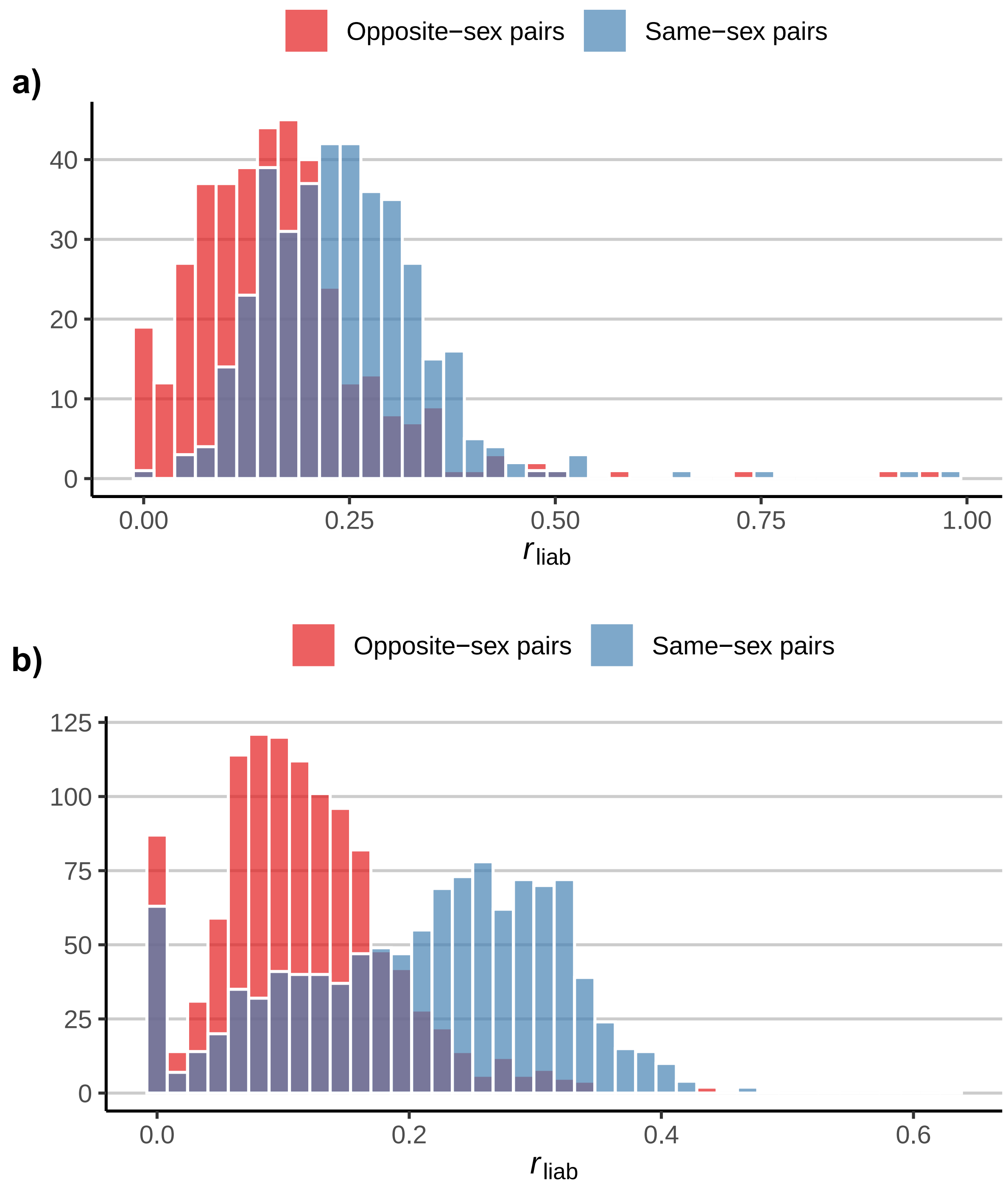

Supplementary Figure 5: Pair correlations on liability scale. Distribution of same-sex (blue) and opposite-sex (red) correlations on liability scale for (a) the twin 1955 cohort and (b) the sibling 1955 cohort.

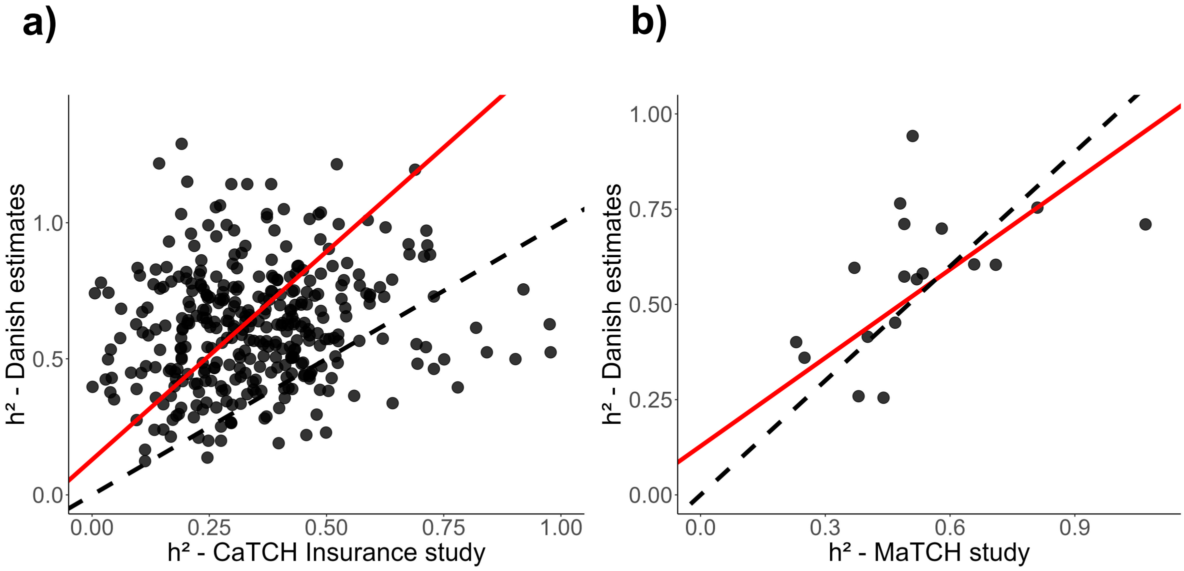

Supplementary Figure 6: Comparison of Danish sibling heritability estimates with previous twin-based studies. (a) Weighted regression comparing Danish sibling heritability estimates to those from the CaTCH study. Each point represents a phenotype with non-zero heritability in both datasets. The red line indicates the weighted linear regression fit, while the dashed black line represents the identity line (slope = 1). (c) Weighted regression comparing Danish sibling estimates with those from the MaTCH meta-analysis.

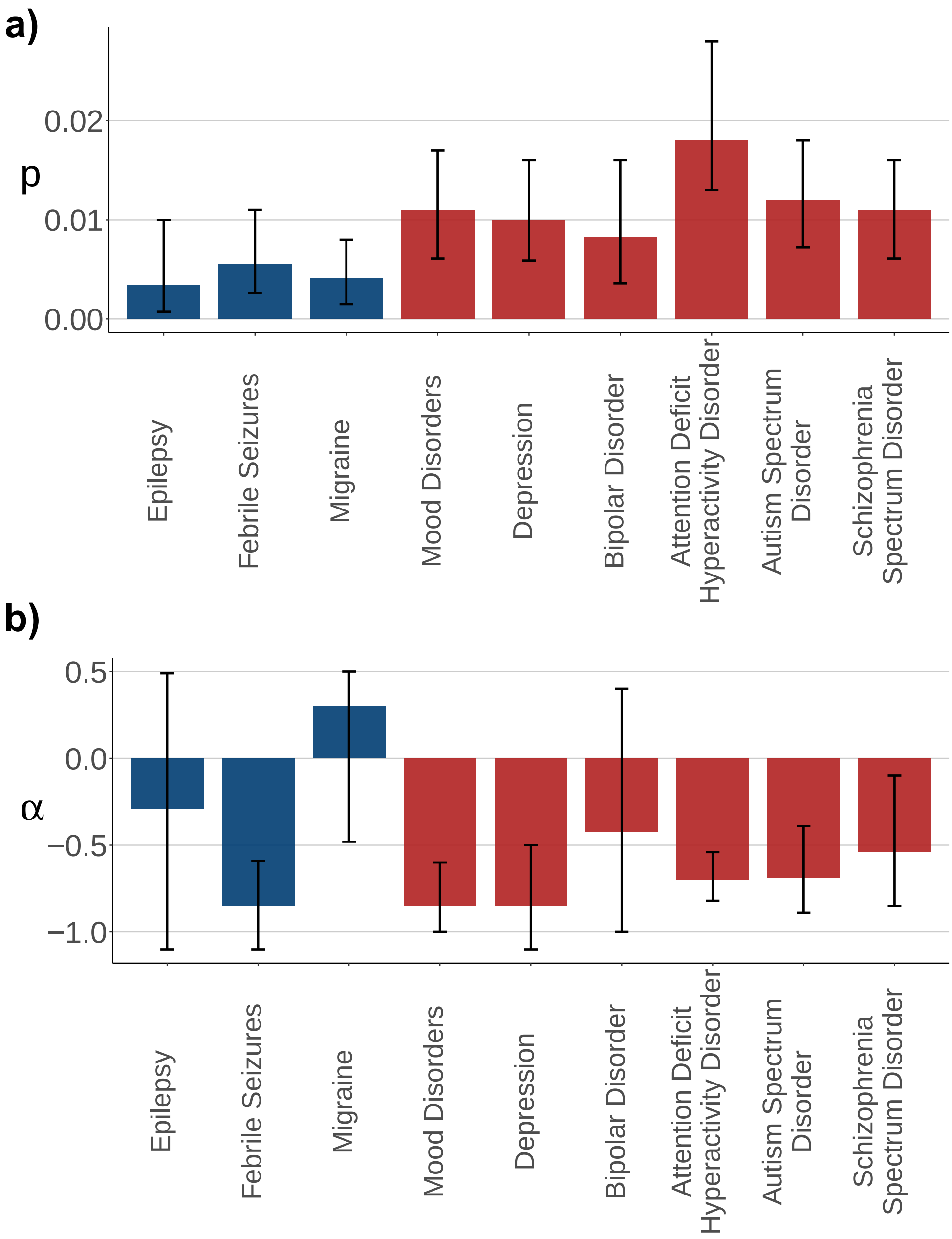

Supplementary Figure 7: Polygenicity (p) and selection coefficients (α) for selected neurological and psychiatric disorders. (a) polygenicity and (b) selection coefficients for neurological (blue) and psychiatric (red) disorders estimated from LDpred2-auto. Error bars indicate 95% confidence intervals.
