# Supplementary material for: Mapping the heritability of disease: a nationwide study
Source: Nat Commun. 2026 Mar 17;17:4080. doi: 10.1038/s41467-026-69991-z (PMC13144670; doi:10.1038/s41467-026-69991-z)
Supplement: Supplementary file 2 — Description of Additional Supplementary Files [file 41467_2026_69991_MOESM2_ESM.pdf]

Description of Additional Supplementary Files

Supplementary Data 1 contains all twin estimates from both the 1955 and 1977 birth cohorts with standard errors, 95% confidence intervals, r_ss and r_os, uncorrected and corrected p values, onset type, and functional domain.

Supplementary Data 2 contains all sibling estimates from both the 1955 and 1977 birth cohorts with the same variables as Supplementary Data 1.

Supplementary Data 3 contains snp-h2, polygenicity, and selection coefficients with 95% confidence intervals for 10 brain disorders.

Supplementary Data 4 contains sex ratios for both the 1955 and 1977 sibling and twin cohorts.
