# Supplementary material for: Mapping the heritability of disease: a nationwide study
Source: Nat Commun. 2026 Mar 17;17:4080. doi: 10.1038/s41467-026-69991-z (PMC13144670; doi:10.1038/s41467-026-69991-z)
Supplement: Supplementary file 7 — Reporting Summary [file 41467_2026_69991_MOESM7_ESM.pdf]

nature portfolio

Corresponding author(s): Janne Auning

Last updated by author(s): Feb 4, 2026

Reporting Summary

Nature Portfolio wishes to improve the reproducibility of the work that we publish. This form provides structure for consistency and transparency in reporting. For further information on Nature Portfolio policies, see our Editorial Policies and the Editorial Policy Checklist.

Statistics

For all statistical analyses, confirm that the following items are present in the figure legend, table legend, main text, or Methods section.

n/a	Confirmed	
☐	☒	The exact sample size (*n*) for each experimental group/condition, given as a discrete number and unit of measurement
☐	☒	A statement on whether measurements were taken from distinct samples or whether the same sample was measured repeatedly
☐	☒	The statistical test(s) used AND whether they are one- or two-sided<br>*Only common tests should be described solely by name; describe more complex techniques in the Methods section.*
☐	☒	A description of all covariates tested
☐	☒	A description of any assumptions or corrections, such as tests of normality and adjustment for multiple comparisons
☐	☒	A full description of the statistical parameters including central tendency (e.g. means) or other basic estimates (e.g. regression coefficient) AND variation (e.g. standard deviation) or associated estimates of uncertainty (e.g. confidence intervals)
☐	☒	For null hypothesis testing, the test statistic (e.g. *F*, *t*, *r*) with confidence intervals, effect sizes, degrees of freedom and *P* value noted<br>*Give P values as exact values whenever suitable.*
☒	☐	For Bayesian analysis, information on the choice of priors and Markov chain Monte Carlo settings
☒	☐	For hierarchical and complex designs, identification of the appropriate level for tests and full reporting of outcomes
☐	☒	Estimates of effect sizes (e.g. Cohen's *d*, Pearson's *r*), indicating how they were calculated

Our web collection on statistics for biologists contains articles on many of the points above.

Software and code

Policy information about availability of computer code

Data collection	No software was used to collect data. Data were obtained from the Danish Civil Registration System and the Danish National Patient Register, linked via unique personal identifiers.
Data analysis	All statistical analyses were conducted using R (version 4.4.1). Linear mixed models were implemented using the lme4 package (version 1.1.35.5) for variance component estimation. Bootstrap confidence intervals were computed using the boot package (version 1.3.30). Heritability estimation followed the CaTCH framework, adapted for register data as described in Lakhani et al. (2019). SNP-heritability, polygenicity, and selection parameter analyses used LDpred2-auto from the bigsnpr R package (version 1.12.18).<br>The code used to perform these analyses is made public at https://github.com/janneah/heritability

For manuscripts utilizing custom algorithms or software that are central to the research but not yet described in published literature, software must be made available to editors and reviewers. We strongly encourage code deposition in a community repository (e.g. GitHub). See the Nature Portfolio guidelines for submitting code & software for further information.

Data

Policy information about availability of data

All manuscripts must include a data availability statement. This statement should provide the following information, where applicable:

- Accession codes, unique identifiers, or web links for publicly available datasets
- A description of any restrictions on data availability
- For clinical datasets or third party data, please ensure that the statement adheres to our policy

Data are not publicly available due to Danish data protection regulations. The study used individual-level data from the Danish Civil Registration System, the Danish National Patient Register, and the iPSYCH case-cohort sample, which are protected under national legislation.

Research involving human participants, their data, or biological material

Policy information about studies with human participants or human data. See also policy information about sex, gender (identity/presentation), and sexual orientation and race, ethnicity and racism.

Reporting on sex and gender	Sex was determined based on administrative records in the Danish Civil Registration System and was used to stratify twin pairs into same-sex and opposite-sex groups for heritability estimation.
Reporting on race, ethnicity, or other socially relevant groupings	Race, ethnicity, and other socially relevant groupings were not recorded or used in this study.
Population characteristics	The study population comprised all twins and full siblings born in Denmark between 1955 and 2021 and identifiable through the Civil Registration System. Participants were linked to diagnostic data from the Danish National Patient Register (1977–2021). Sex and age were included as covariates in all models. For the SNP-heritability, polygenicity, and selection parameter analyses, a subcohort from the iPSYCH study (individuals born 1981–2008) was used, comprising genotyped cases with psychiatric disorders and a population-matched subcohort.
Recruitment	No direct participant recruitment was performed; data were obtained from national administrative registers.
Ethics oversight	The use of the data in this study follows the standards of the Danish Scientific Ethics Committee, the Danish Data Protection Agency, and the Danish Health Data Authority. All procedures comply with Danish legislation on biomedical research using existing biobanks and population registers, under which informed consent is not required. Data access was granted through secure research environments in accordance with institutional approvals and national data protection guidelines.

Note that full information on the approval of the study protocol must also be provided in the manuscript.

Field-specific reporting

Please select the one below that is the best fit for your research. If you are not sure, read the appropriate sections before making your selection.

[x] Life sciences   [ ] Behavioural & social sciences   [ ] Ecological, evolutionary & environmental sciences

For a reference copy of the document with all sections, see nature.com/documents/nr-reporting-summary-flat.pdf

Life sciences study design

All studies must disclose on these points even when the disclosure is negative.

Sample size	Sample sizes were not predetermined by power calculations, as the study used nationwide registry data and included all eligible individuals. The final sample included 56,990 twin pairs and 1,115,260 sibling pairs born between 1955 and 2021, with follow-up data available from the Danish National Patient Register.
Data exclusions	Exclusions were made based on pre-specified criteria to ensure valid heritability estimation. Specifically, we excluded phenotypes with fewer than five concordant same-sex and opposite-sex twin pairs, phenotypes with sex ratios exceeding 5:1 or 1:5, and individuals with missing diagnostic or parental linkage information. These criteria were defined in advance to comply with data protection guidelines.
Replication	Replication was assessed internally by comparing heritability estimates across two independent birth cohorts (1955–2021 and 1977–2021). High concordance between cohorts confirmed the robustness of the estimates. Additionally, heritability estimates were benchmarked against previously published results from the CaTCH and MaTCH studies, demonstrating strong agreement in major domains.
Randomization	Randomisation was not applicable, as the study is observational and based on pre-existing population-wide registry data.
Blinding	Blinding was not applicable. Data were obtained from national registers and analysed using de-identified datasets.

Reporting for specific materials, systems and methods

We require information from authors about some types of materials, experimental systems and methods used in many studies. Here, indicate whether each material, system or method listed is relevant to your study. If you are not sure if a list item applies to your research, read the appropriate section before selecting a response.

Materials & experimental systems

n/a	Involved in the study
☒	☐ Antibodies
☒	☐ Eukaryotic cell lines
☒	☐ Palaeontology and archaeology
☒	☐ Animals and other organisms
☒	☐ Clinical data
☒	☐ Dual use research of concern
☒	☐ Plants

Methods

n/a	Involved in the study
☒	☐ ChIP-seq
☒	☐ Flow cytometry
☒	☐ MRI-based neuroimaging

Plants

Seed stocks

Report on the source of all seed stocks or other plant material used. If applicable, state the seed stock centre and catalogue number. If plant specimens were collected from the field, describe the collection location, date and sampling procedures.

Novel plant genotypes

Describe the methods by which all novel plant genotypes were produced. This includes those generated by transgenic approaches, gene editing, chemical/radiation-based mutagenesis and hybridization. For transgenic lines, describe the transformation method, the number of independent lines analyzed and the generation upon which experiments were performed. For gene-edited lines, describe the editor used, the endogenous sequence targeted for editing, the targeting guide RNA sequence (if applicable) and how the editor was applied.

Authentication

Describe any authentication procedures for each seed stock used or novel genotype generated. Describe any experiments used to assess the effect of a mutation and, where applicable, how potential secondary effects (e.g. second site T-DNA insertions, mosiacism, off-target gene editing) were examined.
